# Distinguishing Self, Other, and Autonomy From Visual Feedback: A Combined Correlation and Acceleration Transfer Analysis

**DOI:** 10.3389/fnhum.2021.560657

**Published:** 2021-09-01

**Authors:** Berkay Demirel, Clément Moulin-Frier, Xerxes D. Arsiwalla, Paul F. M. J. Verschure, Martí Sánchez-Fibla

**Affiliations:** ^1^Artificial Intelligence and Machine Learning Group, Department of Information and Communications Technologies, Universitat Pompeu Fabra, Barcelona, Spain; ^2^Flowers, Inria and Ensta, University of Bordeaux and Paris Tech, Paris, France; ^3^Department of Information and Communications Technologies, Universitat Pompeu Fabra, Barcelona, Spain; ^4^Laboratory of Synthetic, Perceptive, Emotive and Cognitive Systems, Institute for Bioengineering of Catalonia, Barcelona Institute of Science and Technology, Barcelona, Spain; ^5^The Barcelona Institute of Science and Technology, Barcelona, Spain; ^6^Catalan Institution for Research and Advanced Studies (ICREA), Barcelona, Spain

**Keywords:** theory of mind, cognitive development, autonomy, attention, agency, sensorimotor learning, developmental psychology, computational cognition

## Abstract

In cognitive science, Theory of Mind (ToM) is the mental faculty of assessing intentions and beliefs of others and requires, in part, to distinguish incoming sensorimotor (SM) signals and, accordingly, attribute these to either the self-model, the model of the other, or one pertaining to the external world, including inanimate objects. To gain an understanding of this mechanism, we perform a computational analysis of SM interactions in a dual-arm robotic setup. Our main contribution is that, under the common fate principle, a correlation analysis of the velocities of visual pivots is shown to be sufficient to characterize "the self" (including proximo-distal arm-joint dependencies) and to assess motor to sensory influences, and "the other" by computing clusters in the correlation dependency graph. A correlational analysis, however, is not sufficient to assess the non-symmetric/directed dependencies required to infer autonomy, the ability of entities to move by themselves. We subsequently validate 3 measures that can potentially quantify a metric for autonomy: Granger causality (GC), transfer entropy (TE), as well as a novel “Acceleration Transfer” (AT) measure, which is an instantaneous measure that computes the estimated instantaneous transfer of acceleration between visual features, from which one can compute a directed SM graph. Subsequently, autonomy is characterized by the sink nodes in this directed graph. This study results show that although TE can capture the directional dependencies, a rectified subtraction operation denoted, in this study, as AT is both sufficient and computationally cheaper.

## 1. Introduction

We are just beginning to uncover the mysteries of how the brain makes sense of the surrounding world and learns to perform social interactions during development. Babies are very sensitive to motion and use it to organize visual scenes into higher-order structures and seem to rely on instantaneous, immediate motion co-occurrences (Gelman, 2003; Luo et al., 2009). Babies learn to recognize themselves and other agents (Gelman, 2003) and develop a notion of autonomous entities that can actively function by themselves (like persons or animals), or need to be actuated by others (like objects and toys). That is to say, babies learn to distinguish animate from inanimate entities early on Luo et al. (2009) and Opfer and Gelman (2011) (thus we can also say that babies learn to assess whether an entity is autonomous or not). In addition, there is evidence that the brains of vertebrates have ancient neural mechanisms susceptible to the detection of animacy, as they call it in Mascalzoni et al. (2010) study. Learning these distinctions is a basic prerequisite of how babies acquire social cognition (Baillargeon et al., 2015), which is closely tied to the acquisition of a Theory of Mind (ToM), that is, learning to assess and predict beliefs, intentions, and goals of others. Recent developments in AI (based on Bayesian probabilistic inference Baker et al., 2011 and Deep Learning Rabinowitz et al., 2018) have attempted to address the challenge of learning to acquire a ToM, that is assessing and predicting the beliefs, intentions and goals of other agents (refer to also Freire et al., 2018, 2019 for a control theoretic perspective). ToM can be assessed at many levels, but a reliable ultimate test should be able to answer questions about states of the environment of the agent (including other agents), as in Nematzadeh et al. (2018), in which neural models augmented with external memory structures are evaluated in question answering. Indeed, the underlying question is whether cognitive mechanisms underlying action and perception in the physical world can somehow be generalized to the social setting, involving beliefs and intentions of other agents (Arsiwalla et al., 2016, 2017a,b; Verschure, 2016). The long-term goal of this research agenda is to provide a computational basis for how ToM abilities could arise from low-level sensorimotor (SM) interactions, that is, bottom-up from agent interaction behaviors (Freire et al., 2018, 2019). In this sense, we differ from multi-agent approaches like (Marsella et al., 2004), where beliefs are symbols added as logical facts and inference is performed through rule systems. We also distinguish this minimal correlational/temporal low-level SM approach from learning complex parameterized neural nets like (Rabinowitz et al., 2018). Presumably, in a later phase of learning and development, and *via* building up from the bottom up interactions, ToM abilities may be refined *via* top-down optimization of social behavior and cooperation.

Acquiring ToM abilities requires labeling and clustering the SM data stream of the interaction of the agent in terms of which visual features belong to its own body, which ones belong to other entities, which ones can be controlled by its actuators, and which ones can be controlled by themselves or are passive and need others to move. The problem of deciphering self from others in robotics and AI has been addressed by several computational models in studies such as Brody et al. (2017), Thomas et al. (2017), Sánchez-Fibla et al. (2017b), Rybkin et al. (2018), and Pertsch et al. (2018). We approach the labeling problem from the perspective of identifying what are the minimal requirements to distinguish self, other, and autonomous or passive entities from visual feedback alone. Under the Gestalt principle of common fate: “what moves together, clusters together,” we show that a simple correlational analysis of visual pivots' velocities, can suffice to distinguish “self” and “other” (refer to sections 2.4, 3.1). This happens in the long term, as in the short term, the different parts of the same body may not move together as they may be actuated by different joints moving in different directions.

We subsequently introduce an Acceleration Transfer (AT) measure (Sections 2.6, 3.2) targeting the extraction of directional dependencies between visual pivots for the detection of autonomy defined as the capacity of entities to move themselves. Pairwise directional dependencies cannot be captured by correlation analysis. Addressing the detection of autonomy or animacy (as named by Mascalzoni et al., 2010 and widely addressed by the Developmental Psychology literature Luo et al., 2009; Opfer and Gelman, 2011; Baillargeon et al., 2015) can be considered a novelty and a contribution from a computational modeling perspective (refer to section 2.7). To the best of our knowledge, we are not aware of any other research that state what are the minimal computational requirements for detecting autonomy, which we postulate could be the AT among visual motion pivots (our SIPs). Acceleration is directly linked to force (through Newton's 2nd law), and it reflects shorter events in time, compared to velocity. In addition, there are connections to studies providing neurophysiological evidence of acceleration responsive neurons as in Schlack et al. (2007).

**Figure 1:**
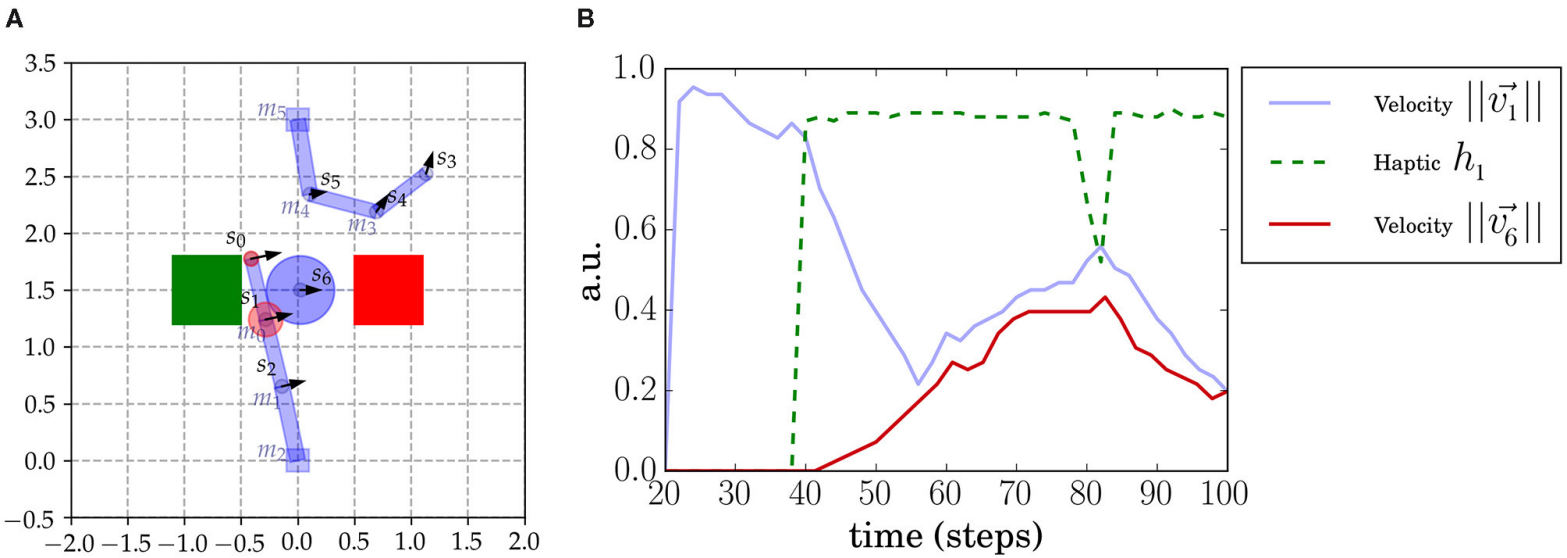
Sally-Anne robot setup. **(A)** Two 3DOF robotic arms face each other and, in the middle, an object is constrained to move in a horizontal axis and cannot be seen when under the boxes (indicated by the green and red squares). The agents perceive the sensory interest points (SIPs) *s*_0_, ..., *s*_6_ placed in the arms and object. Agents also receive tactile feedback when contacts occur (indicated as red circles). We also annotate the different motors (*m*_0_, *m*_1_, *m*_2_ for the lower arm and *m*_3_, *m*_4_, *m*_5_ for the upper arm) placed in each join. The haptic signal of every SIP (that we denote *h*_*i*_) is simulated and computed from proximity to the real contact point (which may be between SIPs). The *h*_*i*_ intensity is depicted with a red circle centered at the corresponding SIP position. **(B)** Sensorimotor time series in a moment of contact of SIPs *s*_1_ and *s*_6_. The haptic signal *h*_1_ (green dashed line) increases suddenly at the moment of contact (at 40 time steps). At this point the velocity of the object increases as well.

The underlying principle of AT measure to detect autonomy is that inanimate entities can only receive acceleration and can never create it by themselves; so, passive entities can accelerate but only through others. It would seem like we are not strictly required to look at pairwise dependencies, but the acceleration of an inanimate object may have been caused by another entity several steps before; so, we need to assess the dependencies of all pairwise interactions. To make fewer assumptions, we face the autonomy identification problem without relying on haptics or filtering by the proximity of visual pivots. Thus, we switch to an alternative underlying principle: inanimate objects will always be a sink in the directional graph of pairwise interactions defined in section 2.8. The so-called, SM graph can be computed from the AT and other standard measures, such as Granger causality (GC) or transfer entropy, although we show that AT has some advantages as discussed in section 3.3. We evaluate and validate both the velocity correlation and AT analysis (with the estimation of directed SM graphs) in a bi-manual, multi-agent, the ball sliding task (freely available^1^, refer to Figure 1 and section 2.2 for details).

## 2. Methods

We define the components that are going to constitute the sensory space and the motor actuators *via* a simulated environment that will emulate a newborn in the presence of passive objects and another agent. We will describe which sensory and motor signals we will consider and we will build a methodology to address how one can minimally extract, and make sense, the SM stream of data. The particularity of this method is that we address this problem solely from the visual modality. But we do not follow a typical deep learning approach (Mnih et al., 2015), instead, we identify the minimal elements from which we can start reasoning about visual motion cues, through visual pivots reminiscent of the biological motion (BM) pivot dots (which are in turn related to social cognition (Pavlova, 2011).

### 2.1. Notation

The visual modality is reduced to a set of relevant sensory interest points (SIPs), as we formalize in this study based on Sánchez-Fibla et al. (2019). Reducing the visual input to a set of SIPs does not seem to be a limiting assumption, as humans and animals, in general, have the ability to understand and recognize action from the observation of a few dots attached to relevant parts of a body in motion, also called BM. BM is also related to social cognition as patients with deficits in social interaction are also compromised in visual body motion processing (Pavlova, 2011).

A SIP is attached to a relevant feature of the visual field (i.e., the end point effector, at the junction between two arm links, at the corner of a square object, etc.). Refer for example the prototypical SIPs that we chose for this setup in Figure 1. SIPs can be computed from a stream of images *via* different methods (including deep learning techniques), but a method that would provide direct mapping is the so-called Scale Invariant Feature Transform (SIFT) points, which provides local feature pivot points (Lowe, 1999).

A sensory state *s* consists of a set of SIPs: *s* = {*s*_0_, ⋯ , *s*_*i*_, ⋯ } each *s*_*i*_ being {*p*_*i*_ = {*x*_*i*_, *y*_*i*_}, *h*_*i*_} where *p*_*i*_ corresponds to its 2D Cartesian coordinates and *h*_*i*_ a real value haptic signal normalized from 0 to 1. The changing signal information for each SIP can be extracted from the temporal SM data: $\Delta s_{i}=\left\{\vec{v_{i}}=\left\{v_{i}^{x},v_{i}^{y}\right\},\vec{a_{i}}=\left\{a_{i}^{x},a_{i}^{y}\right\},\Delta h_{i}\right\}$

Where $\vec{v_{i}}$ and $\vec{a_{i}}$ denotes the velocity and acceleration vector of *s*_*i*_, respectively. We are going to assume that vectors are normalized, $||\vec{v_{i}}||\in \left[0..1\right]$, $||\vec{a_{i}}||\in \left[0..1\right]$. Δ*h*_*i*_ is the haptic signal change. In continuation we will consider and refer to time series of positional information of SIPS (*x, y* coordinates), velocities of SIPs ($\vec{v}$ velocity vector with *v*^*x*^, *v*^*y*^ components and magnitude $\left||\vec{v}|\right|$), and accelerations ($\vec{a}$ acceleration vector with *a*^*x*^, *a*^*y*^ components and magnitude $\left||\vec{a}|\right|$).

The motor apparatus of a newborn includes over 650 skeletal muscle actuators. We restrict this study to a small number of actuators. A motor state *m* = {*m*_0_, ..., *m*_*n*−1_} corresponds to the angles of every joint *m*_*i*_ ∈ [0...2π]. The motor space is denoted by M. A delta motor state Δ*m* = {Δ*m*_0_, ..., Δ*m*_*n*−1_} is the velocities of each joint, *n* being the total number of DOFs.

### 2.2. Implementation of the Sally-Anne Paradigm

The so-called Sally-Anne setup is a robotics implementation (depicted in Figure 1A) inspired from the psychological experiment with the same name, designed to probe attribution of beliefs and was first studied in relation to autism (Baron-Cohen et al., 1985) (although the result stating that autistic subjects fail at the “Sally-Anne test” is still under debate Tager-Flusberg, 2007). Let us briefly summarize the experiment. The participant is presented with two boxes and an object and an imaginary character, Sally, places the object in box A and leaves the room. Another imaginary character, Anne, puts the object in the other box B. Sally comes back and the participant is asked, in which box will Sally look for the object? The test is passed if box A is chosen, as, although we know that the object is in box B, Sally cannot know it because she did not see Anne transferring the object to the other box. This paradigm thus probes the ability of the participant to model belief states of the other.

This setup here, inspired by the Sally-Anne experiment, is a synthetic computational implementation of the Sally-Anne paradigm. It consists of two agents (two robot arms), an object and two colored boxes or areas that only occlude the entities underneath (refer to Figure 1A). The two-arm robots face each other so that there is a region in the space where both interact, thereby affecting each other's actions. Depending on the current state, each agent can move freely, interact with the object alone, interact with the other agent, or interact with the other agent using the object. The object is a sliding ball that is constrained to move in an horizontal line between the two boxes. Each entity in the setup has associated sensory points (attached to joints in the robot arms and the object). In total, seven SIPs are considered: three in each arm of the robots and one in the object. End effector SIPs are denoted by *s*_*left*_ = *s*_0_ and *s*_*right*_ = *s*_3_. A SIP is added at the object: *s*_*obj*_ = *s*_6_ at the center. Each agent perceives the totality of the visual cues of the scene (all SIPs, from *s*_0_ to *s*_6_, the object) and their haptic signals. Each robot arm has three joints that can be actuated independently by each agent.

The haptic signals in this setup are simulated and are computed considering their distance to the closest contact point (in Figure 1A haptic signals are represented by red circles centered at its SIP). The haptic signal *h*_0_ seems to increase inconsistently, but, as shown in Figure 1A, *h*_0_ is faint on initiation of contact and increases slowly because it gets closer to the contact point between the two shapes (rectangle and circle). On the other hand, *h*_1_ is closer to the real contact point so it increases rapidly, as shown in Figure 1B.

### 2.3. Data Generation

Sensorimotor data is generated from a mixture of behaviors: motor babbling and goal-directed movements targeting the object. Motor babbling assigns random velocities to the motor joints of the arms (Δ*m*_*i*_ for *i* ∈ 0, 1, 2 for the bottom agent and *i* ∈ 3, 4, 5 for the top one) at different moments. We need independent motor babbling to not cause an artificial correlation between the agents due to the synchronous randomly generated behavior. We sustain, for different time steps, the assigned motor torque to each joint *m*_*i*_. We need to sustain the torque applied to generate a consistent movement with a consistent effect during a certain time. Because of the characteristics of the setup, we needed to set certain rules on top of the independent random signals sent to the motor joints to generate a rich and representative dataset. For example, when the object reached an end-point under one of the two boxes, the object was reset in the middle again, and the two arms were positioned at their initial positions. As an example, in Figure 1B, we show the time series of *s*_1_ and *s*_6_ SIPs before and during an object contact.

Generating the behavior of agents in this way, we acquire a dataset of all SIP data streams (position, velocities, accelerations, and haptic contacts) and we are thus ready to apply the corresponding statistical tools to determine the nature of every SIP.

### 2.4. Correlational Analysis

We start by presenting a minimal method to assess which elements of the visual sensory scene correspond to the self and the rest (other entities, objects, or other agents). We do so by a correlational analysis of the different streams of data from the generated dataset. The Pearson's correlation (or simply correlation) coefficient accounts for the linear relationship between a set of points with (*x, y*) coordinates. In general, these coordinates are the outcome of two random variables. In this case, we look at the correlation between SIP measures, like *x* coordinates, velocities, accelerations, and we also consider their haptic feedback values. The correlation between two random variables *X* and *Y* is then defined by:

$$\rho_{X,Y}=\text{corr}\left(X,Y\right)=\frac{\text{cov}\left(X,Y\right)}{\sigma_{X}\sigma_{Y}}=\frac{𝔼\left[\left(X-\mu_{X}\right)\left(Y-\mu_{Y}\right)\right]}{\sigma_{X}\sigma_{Y}}$$

where μ_*X*_, μ_*Y*_ are the respective variable means and σ_*X*_, σ_*Y*_ are the SD or variances. Correlation is undefined when either one of the variances is 0 (division by zero). This happens, for example, when one variable is a constant value. This can happen in this case when looking for correlations of the end-point of an arm *s*_0_ and the object *s*_6_, in the case where the object remains static and untouched.

We aim to stick with the simplest methods possible to extract self/other/object characteristics from SM data. But, of course, correlation comes with its limitations. First, Pearson's correlation captures the amount of linear relationship between two variables and can have some trouble in detecting dependencies that are not strictly linear, like the ones between motor signals *m*_*i*_ and sensory point coordinates of SIPs *s*_*i*_. Second, correlation does not allow us to infer temporal or causal relationships between sensory events. A correlational analysis does not provide directional information as the Pearson correlation coefficient is symmetric: corr(*X, Y*) = corr(*Y, X*).

### 2.5. Temporal Dependencies

In the context of correlational analysis, the one obtained by computing corr(*X, Y*), from data time series extracted from SIPs, the temporal structure of data is completely discarded. The sampled values of variables *X* and *Y* have no temporal relationship and they are only inspected in pairs at the same time point *t*. There are many ways of considering temporal (and order) information. The main principle used when doing this is to assess what a second-time series adds to the prediction of another one. What is the added prediction value of time series *x*^0..*n*^ to time series *y*^0..*n*^? Consider the sample at time *t*, *y*^*t*^. How the samples *x*^*t*−*i*^ help to predict *y*^*t*^ in addition to *y*^*t*−*i*^.

Let *x*^0...*n*^ and *y*^0...*n*^ be stationary time series. *x*^0...*n*^ is said to Granger cause *y*^0...*n*^ if it has an added value in the prediction of *y*^0...*n*^. If values of *x*^0...*t*−1^ add explanatory power to *y*^*t*^ (in addition to *y*^0...*t*−1^), *x*^0...*n*^ is said to Granger cause *y*^0...*n*^.

Granger causality is based on a linear correlation test between past and current values, implemented with a statistical *t*-test. We based all our previous analysis on correlations, which only capture linear relationships, and these were sufficient to extract the proximo-distal and motor to sensory relations. It seems a perfectly logical approach to use GC to capture directional relationships.

Transfer Entropy (Vicente et al., 2011) can deal with non-linear relationships, and many indicate that it is more suitable for quantifying causality (Razak and Jensen, 2014). TE comes with its drawbacks: taking into account just a small-time history, necessary for its computation, it becomes inefficient to compute and demands a lot of data to have maximum exposure to all history combinations.

Both measures, GC and TE, have an additional parameter which is the lag or history time steps that the computation takes into consideration. The lag is an additional parameter that in the case of TE is problematic as it demands an increasing exponential number of samples to be accurate (as the history increases).

Granger causality and TE are based on distribution of the sampled data, and we need a measure to operate on a trial-by-trial basis. Thus, we cannot ensure stationarity of the time series when dealing with isolated trials. Refer to **Figure 5** for example, in which we show a single approach and contact trial of one arm and the object. Although we can make linear piece-wise relations (using the modulus/magnitude of velocity and acceleration vectors), the effect detection mechanism that we are looking for is not linear as a whole. When two SIPs enter in contact, there is first an anti-coupling followed by a progressive coupling after contact (we explain this in more detail in continuation).

### 2.6. Acceleration Transfer Measure

To overcome some of the drawbacks of GC and TE (as discussed in previous section 2.5) which also hold for other measures, such as Copula-Based measure (Junker et al., 2019), we define an alternative instantaneous AT measure. By instantaneous and not temporally dependant we mean that the AT measure does not have a lag/history parameter because it can be computed at a single time step. The AT measure is computed according to an instantaneous positive subtraction of accelerations between two SIPs. Its computation follows the intuition that if a SIP is losing acceleration it can be because it is being transferred to another SIP. As Newton's law states (*F* = *m*.*a*), acceleration is proportional to force, meaning that AT can be seen as a form of force transferred or being applied to.

Consider $a_{i}^{0...t...n},a_{j}^{0...t...n}$ to be the sampled accelerations of SIPs *i* and *j* from time step 0 to *n*. Then, the AT transfer from SIP *i* to *j* at time *t* will simply be the subtraction of accelerations at time *t*: $T\left(a_{i}^{t},a_{j}^{t}\right)=a_{j}^{t}-a_{i}^{t}$. We could then consider the AT between the two SIPs $A_{v0}\left(a_{i},a_{j}\right)$ to be the sum over all the time steps $A_{v0}\left(a_{i},a_{j}\right)=\overset{t}{\underset{k=0}{\sum }}T\left(a_{i}^{k},a_{j}^{k}\right)$. We are going to consider $A_{v0}$ to be the simplest version 0 of the measure. We are then going to add three filters that will target the AT events that we want and will constitute versions 1, 2, and 3. The final AT measure will contain all filters and will be considered version 4.

- Version 1. We are going to filter the instantaneous transfer by the minimum of the two signal values, that is, the transfer cannot be bigger than the minimum of the two signals. The intuition behind this filter is that if signals are very different in magnitude we should be careful not to allow a too big transfer also because the signals may not be related.
  $$\begin{array}{l} T\left(a_{i}^{k},a_{j}^{k}\right)=\{\begin{array}{ll} min\left\{a_{j}^{k}-a_{i}^{k},min\left\{\left|a_{i}^{k}|,|a_{j}^{k}\right|\right\}\right\} & \text{if }a_{j}^{k}-a_{i}^{k}>0\\ max\left\{a_{j}^{k}-a_{i}^{k},-min\left\{\left|a_{i}^{k}|,|a_{j}^{k}\right|\right\}\right\} & \text{otherwise} \end{array} \end{array}$$
- Version 2. If one signal is close or equal to 0, then, the instantaneous transfer is also set to 0. This filter can be enforced by adding a multiplying factor to the instantaneous transfer $T\left(a_{i}^{k},a_{j}^{k}\right)=\left(a_{j}^{k}-a_{i}^{k}\right)*min\left\{1,|a_{i}^{k}||a_{j}^{k}|\right\}$
- Version 3. Signals should have opposite signs. We consider only transfers in which one signal is losing acceleration while the other is gaining it. This is because we are interested in collisions and moments of energy exchange. We enforce this filter with the following inequality that needs a small epsilon (i.e., ϵ = 0.01) to filter out signals of the same sign:
  $$\begin{array}{l} T\left(a_{i}^{k},a_{j}^{k}\right)=\{\begin{array}{ll} a_{j}^{k}-a_{i}^{k} & \text{if }|a_{i}^{k}|+|a_{j}^{k}|\geq |a_{i}^{k}+a_{j}^{k}|+\epsilon \\ 0 & \text{otherwise} \end{array} \end{array}$$

The AT measure that we use (version 4) includes all filters. We analyze the effects of the three filters in the Results section 3.2 applied to different synthetic generated signals.

The AT measure can be refined further using filters of visual proximity or haptic signals from SIPs *h*_*i*_, with the additional assumptions that there can not be a transfer of acceleration between two SIPs that are not touching or that are not in close visual proximity of each other. We prefer to address the more general setting by limiting the number of underlying assumptions, and focusing on showing the viability of this measure as a computationally cheaper and sufficient alternative rather than a specific implementation. Haptic signals as well as visual proximity are neverthesless strong canditates for additional assumptions in developmental settings and can be the basis of further research.

### 2.7. Detecting Autonomy

We define the autonomy (of an entity) as the ability to move by itself. In the setup that we present, two autonomous entities constitute the lower (with associated SIPs *s*_0_, *s*_1_, *s*_2_) and upper (associated SIPs *s*_3_, *s*_4_, *s*_5_) arms and both are controlled by independent motor signals (*m*_0_, *m*_1_, *m*_2_, and *m*_3_, *m*_4_, *m*_5_ respectively). The object and its associated SIP *s*_6_ is a passive entity, non-autonomous, it cannot move by itself but at certain moments it can appear to be moving without any contact (in a low friction environment for example). Thus, the main characterizing property of a passive entity is that it never starts moving without a previous contact, in other words, it never accelerates by itself: an active entity must inject kinetic energy into it. To assess autonomy we need to go beyond correlation and distinguish which SIPs would be dead-ends in the path of directed relations.

### 2.8. The Sensorimotor Graph

Sensorimotor graphs can be constructed in different ways. For instance, in the context of the Distributed Adaptive Control (DAC) (Duff et al., 2011), a directed-graph network stores a compacted version of past sequences of experiences. In the graph, nodes are SM couplets including the reward of the experience of the agent and edges are reinforced when experiences have co-occurred. Other approaches consider SM graphs as memory structures: Toussaint (2006) presents a similar SM directed-graph network approach.

In this study, we consider a SM graph of a different kind. The visual features accessed through the so-called Sensory Interest Point data (SIP coordinates, velocities, accelerations, haptic signals) are considered random variables, and the graph corresponds to the dependencies between these variables. Nodes of the graph are SIP-related variables and edges are extracted dependencies between them (correlations for instance). Indeed, the matrix of pairwise correlations can be binarized using a threshold, thus obtaining an adjacency matrix defining an undirected graph as correlation is symmetric. Using AT, we can produce an asymmetric matrix thus leading to a directed graph. The same is true for any other measure like GC and TE. From their matrices of pairwise dependencies (that we explain in section 3.3), one can extract a directed SM graph.

The directed SM graph can be the fingerprint of autonomy as an inanimate entity should not be capable of transferring acceleration to other entities; thus a non-Autonomous entity will always appear as the sink of a directed SM graph. An inanimate/non autonomous/passive entity is characterized as being the end node in the directed SM graph of the SIP AT measure. There might be rare occasions where this assumption is violated, such as in the case of a billiard ball, where we transfer acceleration to the white ball, which in turn transfers acceleration to other balls, but always losing energy, never gaining it.

## 3. Results

In the current section we present numerical results obtained from the SM data set that we extracted from the Sally and Anne setup (explained in section 2.3). We first present a descriptive interpretation of the correlation analysis of the Sensory Interest Point (SIP) interactions (refer to section 3.1 as seen previously in Sánchez-Fibla et al. (2017b). From the correlation limitations described, we provide results (section 3.2) of the newly introduced AT measure (Methods section 2.6) along with a comparison of the measure with GC and TE measures. The SM Graphs are alternative ways of visualizing the pairwise interactions between SIPs according to the different measures considered.

### 3.1. Correlational Analysis

**Figure 2:**
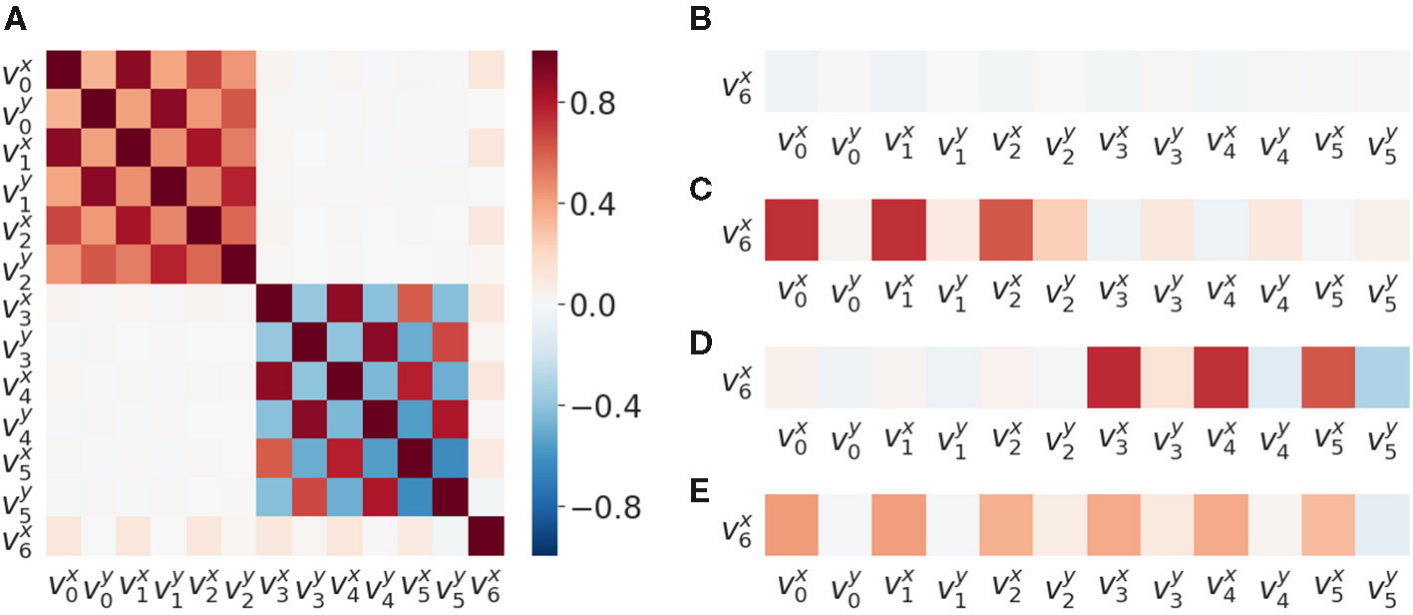
Velocity correlation matrices of SIPs.. **(A)** Velocity correlation matrix of all the SIPs generated from 50,000 random SM interactions. **(B)** Correlation between SIP velocities, object velocity $v_{6}^{x}$ and arms $v_{0}^{x},...,v_{5}^{x}$, when there is no contact (no haptic signal active). **(C)** Correlation between object and arms SIP velocities when there is contact between the lower arm and the object. **(D)** Correlation between object and arms SIP velocities when there is contact between the upper arm and the object. **(E)** Correlation object and arms SIP velocities when there is contact between the object and either arm.

We found that patterns of correlations arise between velocities of SIPs (Figure 2A). A signature of the proximo-distal organization of the arm of each agent is present. The closer the two joints are, the greater their correlation is: $\text{corr}\left(v_{0}^{x},v_{1}^{x}\right)$ is less than $\text{corr}\left(v_{1}^{x},v_{2}^{x}\right)$ for the bottom agent and $\text{corr}\left(v_{3}^{x},v_{4}^{x}\right)$ is less than $\text{corr}\left(v_{4}^{x},v_{5}^{x}\right)$. This inter agent correlation (proximo-distal) pattern is nearly identical between the two agents. Both include the same chessboard pattern as observed in Figure 2A top-left and bottom-right (excluding *v*_6_ row and column which corresponds to the SIP in the object). By matching their own joint velocity correlation pattern with the one observed from another agent, this could provide a first level of mirroring between agents, where each one is able to match its kinematic structure with that of the other.

We also observed a correlation of the object velocity $v_{6}^{x}$ with both arms having different intensities following the same proximo-distal pattern under different conditions: (i) when not in contact (Figure 2B), (ii) when in contact with the lower arm (Figure 2C), (iii) when in contact with the upper arm (Figure 2D), and (iv) when in contact with either one arm or the other (Figure 2E). This pattern may also provide a second level of mirroring, where agents can discover that similar movements of the other provide similar effects on the objects, paving the way to a notion of shared affordances, useful for joint action planning.

**Figure 3:**
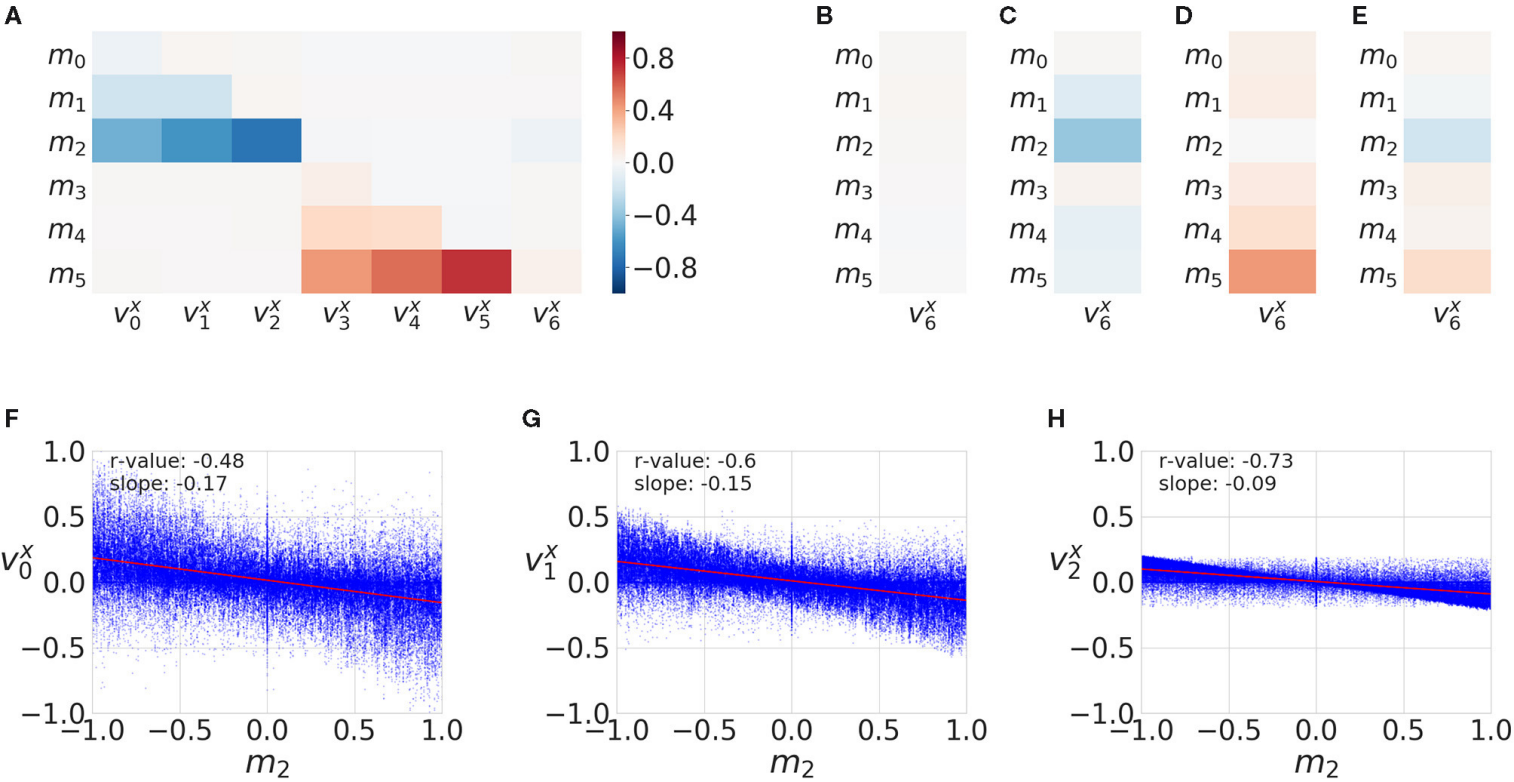
Motor and sensory correlations. **(A)** Correlations matrix between motor activation (*m*_0_...*m*_5_ signals) and SIP velocities *v*_0_, ...,*v*_6_, generated from 50,000 random SM interactions. **(B)** Correlation column of all motor activations with the object SIP velocity $v_{6}^{x}$ for SM data when there is no contact (no haptic signals active) **(C)** Correlation of motors with object SIP velocity when there is contact between the lower (bottom) arm and the object. **(D)** Correlation of motors with object SIP velocity when there is contact between the upper arm and the object. **(E)** Correlation between motor and object SIP when there is contact between the object and either arm. **(F–H)** All data points and linear fit for *m*2 and SIP velocities $v_{0}^{x}$, $v_{1}^{x}$, $v_{2}^{x}$. The *r*-values correspond to the equivalent correlation matrix color values.

We found motor signals to be correlated with velocities of SIPs (Figure 3A). The bottom joint of the bottom agent (*m*_2_) is strongly negatively correlated with the *x* velocity components of sensory points *s*_0_, *s*_1_, and *s*_2_, because this joint moves the whole arm. The same happens with the top agent. This matrix can assess controllability characteristics and potentially restricts the forward model to be learned for the relevant SIP signals: i.e., the agents could filter out SIPs that are not correlated to its available motor signals.

The object *x* velocity component $v_{6}^{x}$ does not appear to correlate with any motor signals (Figure 3A) because, in this study, we consider the totality of the interaction (with and without contact). For this reason, in Figure 2 we distinguish the different conditions with regard to haptic signals: (i) when no contact is made (Figure 3B), (ii) when contact with the lower arm is made (Figure 3C), (iii) when contact with the upper arm is made (Figure 3D), and (iv) when contact with either arm is made (Figure 3E). This motor to object correlation (when in contact with each arm) is reminiscent of the notion of affordance (Sánchez-Fibla et al., 2011), as it characterizes the effect of a motor action into a movement characteristic of the object.

In addition, the correlations change sign in the *x* coordinate for the top and bottom arms. This is because the arms are initialized to different positions and one has a tendency to touch the object in one direction and the other in the opposite one.

Summarizing the correlational velocity analysis captures well the principle of ‘"what moves together clusters together” on the long term and is capable of distinguishing self/other (proximo-distal relations) and motor to sensory dependencies.

### 3.2. Acceleration Transfer

**Figure 4:**
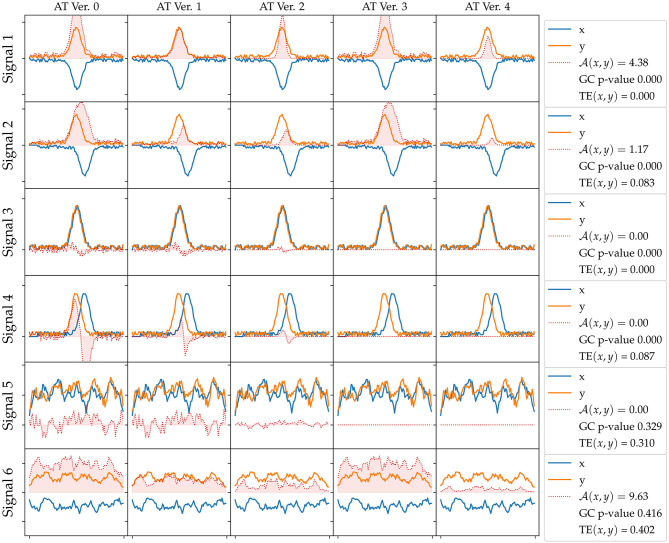
Acceleration Transfer (AT) measure versions evaluated on different synthetic generated signals. The rows correspond to different generated signals: 1) two Gaussians of opposite signs, 2) two Gaussians of opposite signs with a lag, 3) two Gaussians of the same sign, 4) two Gaussians of the same sign with a lag, 5) random smoothed signals, 6) random smoothed signals of opposite signs. The columns correspond to the different versions of the AT (refer to text for further details): V0) signal subtraction, V1) filter by minimum value, V2) filter by closeness to 0, V3) filter by value sign, and V4) all Filters included. In the legend at each row, we show the AT value, the Granger Causality (GC) *p*-value, and transfer entropy (TE) for each signal. A GC *p* < 0.05 means *x* signal Granger causes *y*. TE is in bits.

**Figure 5:**
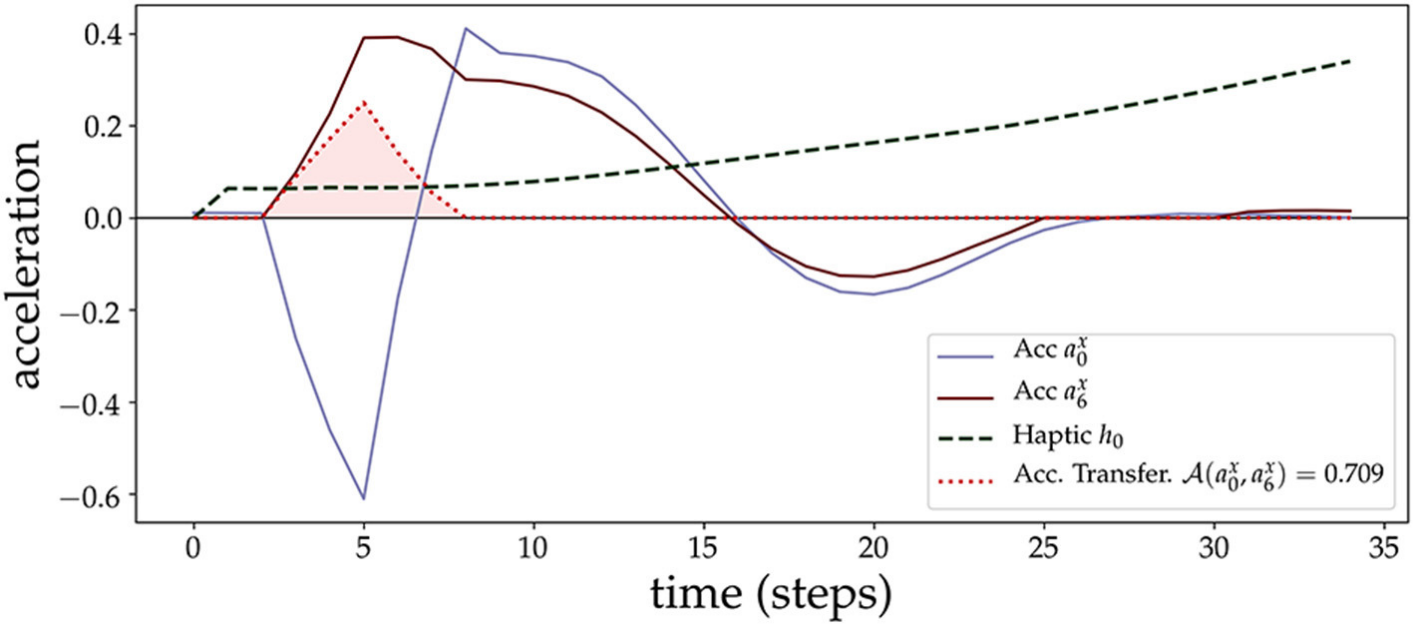
Acceleration Transfer measure. We plot an example of the AT measure (shaded area in red) due to a contact of the lower arm (sensory point *s*_0_, with corresponding acceleration $a_{0}^{x}$) with the object (sensory point *s*_6_, with corresponding acceleration $a_{6}^{x}$). At the beginning of the contact, the arm loses acceleration rapidly which is transferred to the object. After some time steps (after time step 10), both start to couple and synchronize.

We present results of the AT measure introduced in section 2.6, which computes an integration in time of instantaneous acceleration transfers, that is a subtraction of accelerations at a given time step plus a series of filters explained in section 2.6. We plot in Figure 4 an evaluation of all versions of the AT measure (columns) for different generated signals (rows), which all include a small normally distributed noise. In the first row, we plot two signals that include a Gaussian of opposite signs. The physical interpretation of these signals could be the end-point of an arm making contact with the object. The object would then gain acceleration (*y* signal in the figure) and the end-point of the arm would lose it (*x* signal). These signals can be considered a crude approximation of AT from an animate object to an inanimate object (refer to Figure 5 explained below). In the second row, we add some lag to one of the signals emulating some sort of compliance or delayed actuation. We observe that the transfer starts to fade away with a greater lag. In the third row, we plot two Gaussians of the same sign. A possible physical interpretation is that two SIPs are hit by a third one providing energy to both. It makes sense to filter out this situation as it does not correspond to an exchange of acceleration (done by the filter introduced in Version 3). The fourth row shows a variation of the previous situation where there is a lag between the two same signed signals. Fifth row depicts two random signals of the same sign. The last row of Figure 4 reveals a weakness of the AT measure as it may detect continuous false-positive transfers from random signals of opposite signs. These random oscillations are not typical of the minimal jerk movement trajectories of human behavior or the ones that one can program in a robot arm.

We continue with a prototypical example of an arm approaching and making contact with the object. Figure 5 shows an example of the AT measure and how it captures the transfer of acceleration from the lower arm (SIP *s*_0_) to the object (SIP *s*_6_). At the moment of contact, SIPs from the arm (acceleration time series *a*_0_) and the object (acceleration *a*_6_) anti-correlate, giving rise to the moment of acceleration transfer, and then synchronize just afterward, both tending to 0 after the force of the initial contact is exhausted and they continue to move at a constant velocity. AT is 0 in this last part because of the filter applied in version 3 that discards transfers of the same sign. AT is 0 before contact and at the end because of the filter applied at version 2 that sets to 0 the transfer if one of the two signals is close to 0. The different version filters of the AT measure are explained in section 2.6.

**Figure 6:**
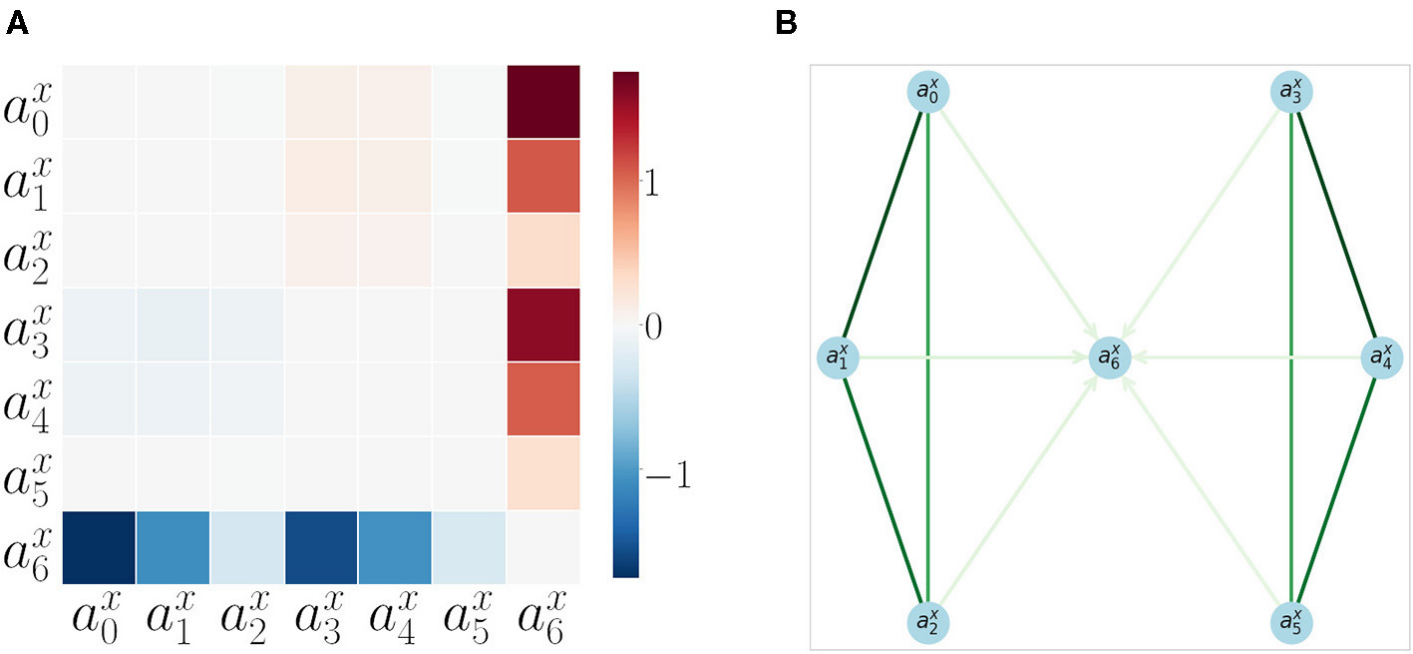
Acceleration Transfer results **(A)** Matrix of pairwise AT measure values between all SIPs for all SM interaction data. **(B)** Directed graph of SIP accelerations $a_{0}^{x}$, .,$a_{6}^{x}$, generated from 200 repetitions of sensorimotor interactions. The edge colors show the correlation strength between the nodes (extracted from the velocity correlation analysis, refer to section 3.1), while the arrows show the direction of AT (refer to section 3.2). Only arrows with a significant AT are plotted. Take note that the node $a_{6}^{x}$ is the only terminal node, as it can not accelerate by itself and depends on the AT from other SIPs.

We also computed the pairwise AT measure (defined in section 2.6) between all acceleration *a*_*i*_ signals. The results are shown as a matrix in Figure 6A. The matrix is computed from 200 repetitions of a random moving arm (with sustained movements for a random number of steps) and the other arm performing a goal-directed approach to the object. Each trial, the arm that moves is chosen randomly with a 0.5 probability. The AT measure with its filters (refer to section 2.6) becomes very specific, and it is able to catch the events we are interested in, which are the object interactions. AT does not detect any interactions between the arms SIPs.

When the AT is applied to the SM data, that is gathered with motor babbling, it usually happens that two SIPs accelerate and decelerate synchronously, and, thus, AT detects lots of false positives. In addition, if random movements are not sustained in time (jerky movements), the AT measure detects lots of false positive transfers as predicted by the random synthetic signals analysis of the last row in Figure 4.

The results are also shown in Figure 6B as a directed graph: the strength of the arrows comes from the previous correlation results (as AT detects no specificity between arms). The direction of the arrows comes from the AT measure. No arrows are plotted for inter-arm relations as AT is not able to capture them. The object appears as the end node (the sink) of the directed graph, proving that it always receives acceleration and never produces it: the signature for a measure of autonomy.

### 3.3. Granger Causality and TE

As discussed in section 2.5, GC requires stationarity, i.e., the mean and variance of each time series do not change over time), and that it can be adequately described by a linear model. We did the Dickey-Fuller test to check for stationary, and it affirmed that all signals (motor activation, velocities, accelerations) are stationary, but not all are linear, especially velocities and accelerations (as both have x and y components). Although GC itself is a linear measure, other non linear versions of causality have also been introduced (Marinazzo et al., 2008). Like standard (linear) GC, non-linear Granger measures are also potentially prone to overfitting and finding false positives. The kernel-based non-linear GC measure in a study by Marinazzo et al. (2008) argues that it solves these two issues and may potentially be a useful measure for this analysis. Similarly, quantum probability methods have been used for cognitive modeling, making extensive use of techniques similar to the kernel-based GC method (see Pothos and Busemeyer, 2013 for a general overview). However, besides the linear/non linear issue, GC also has another problem, that it is inadequate for situations where there are instantaneous effects, as is the case for our experiment here. This is further elaborated on pages 207–208 by Peters et al. (2017): “Knowing that a system contains instantaneous effects may suggest modifying GC by regressing not only on the past, but adding the current value, possibly leading to wrong bidirectional casual influences.” This is in line with our observations.

With respect to TE (Vicente et al., 2011), it is advantageous when the model assumption of GC does not hold, for example, analysis of non linear signals. Thus, it makes sense as a logical next step from GC; however, TE still requires a lot of data and is not suitable for real-time inference. There are suggestions for significantly faster measures of TE based on permutation entropy (Bandt and Pompe, 2002). Although this method may improve over TE, it is not immediately clear whether it can outperform the AT measure, as it requires performing permutation analysis and comparison which is computationally expensive.

Two different sets of experiments were carried out to compare AT, Granger Causality (GC) and TE. In the following paragraphs, the results of the comparison experiments using the acceleration values of the SIPs are summarized^2^. In the first set of experiments, both GC and TE values were calculated on the generated signals explained in section 3.2. In the second set of experiments, all three measures were treated as binary classifiers of directional causality, and their performance tested on the experimental setup described in section 2.2 with increasingly more complex arm policy combinations.

Figure 4 summarizes the results of the first set of experiments, comparing the results of each measure for each signal set. For the first four signals, GC returns a *p*-value below 0, indicating *x* is Granger causing *y*, while for the last two signals GC did not find an effect. TE measure did not find an effect for the first and third signals, where the signals are not lagged, but does find a small entropy transfer for the lagged second and fourth signals regardless of the signal signs. TE finds strong effects in the last two signals, where the signals are random noises. The last version of the proposed AT measure only finds an effect on signals with opposite signs, Signal 1, 2, and 6, effect size decreasing with the lag amount.

**Figure 7:**
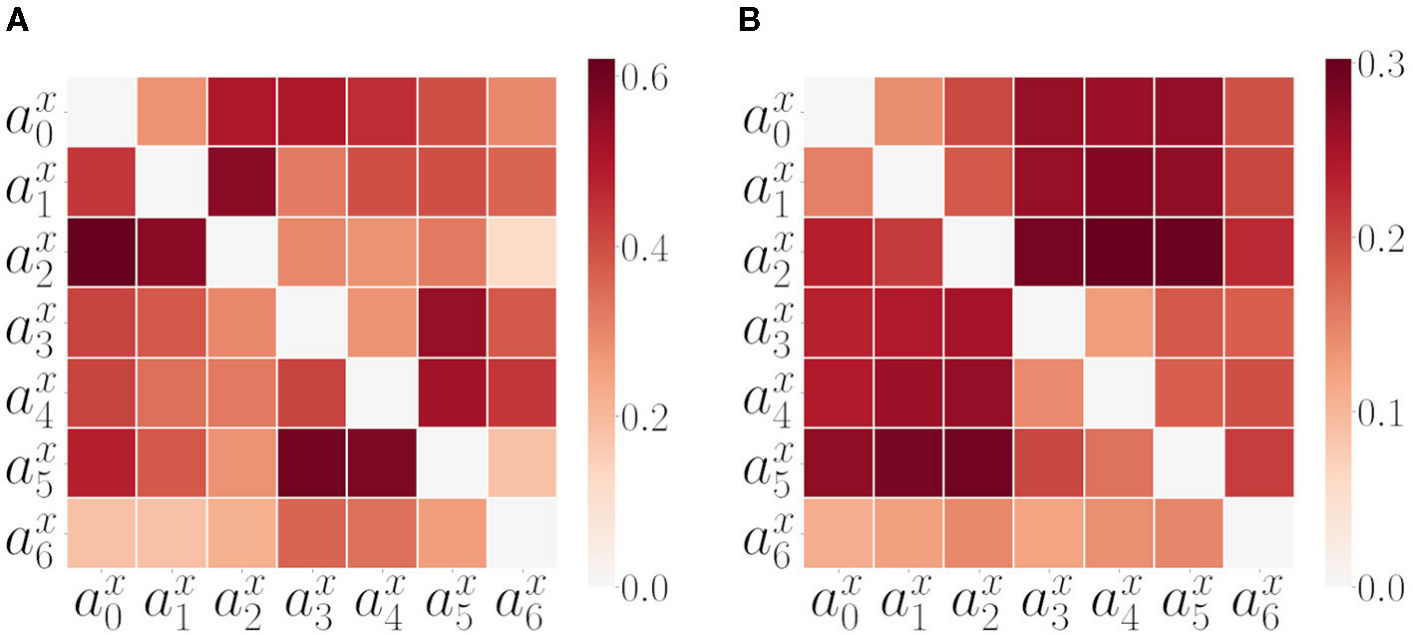
Alternative measures: GC and TE. **(A)** Matrix of pairwise GC measure values among all SIPs for all SM interaction data. The matrix is computed considering the proportion of GC tests passed (refer to text for further details, section 3.3). **(B)** Matrix of pairwise TE measure values among all SIPs for all SM data.

Before executing the second set of experiments, we tested GC and TE on the experimental setup explained in Section 2.2 using a random policy for both arms. In Figure 7 we plot the GC and Transfer Entropy matrices of all pairwise acceleration signals. Both are computed from the same SM data as in Figure 6 extracted from 200 repetitions of a combined random behavior (as discussed in previous section 3.2). The GC matrix that we call, in this study, *GC*^*mat*^ (Figure 7A) is computed considering the proportion of passed GC tests (whenever GC($a_{i}^{x},a_{j}^{x}$) returned a *p* < 0.05). Part of the pattern present in the last column of the AT matrix is maintained in the GC matrix. Also the within arm dependencies are well captured, but we are not interested on those as they are already captured by the correlation analysis. In addition, we can also look at the directional dependencies by comparing $GC^{mat}\left(a_{i}^{x},a_{j}^{x}\right)$ to $GC^{mat}\left(a_{j}^{x},a_{i}^{x}\right)$, but here again, we got inconsistent results. TE matrix (Figure 7B) consistently gives a higher transfer in the direction from the arms to the object, with strong effects for the inter-arm dependencies.

Only considering the case with two random policy arms did not give enough information to make a fair comparison between the measures, leading to the second set of experiments. To assess the performance and limitations of each measure, seven scenarios with increasingly complex policy combinations were prepared:

- Scenario 1: Constant push and static
- Scenario 2: Staggered push and static
- Scenario 3: Random push and static
- Scenario 4: Random and static
- Scenario 5: Random and constant push
- Scenario 6: Random push and constant push
- Scenario 7: Random and random

The constant push policy consists of the arm pushing toward the ball with a constant motor activation, while the staggered push policy consists of the arm either moving with a constant motor activation or no motor activation with 0.5 probability each. The random push policy means the arm moves with variable motor activations sampled from a uniform distribution between 0 and 1. The random policy is the arm moving with motor activations sampled from a uniform distribution between –1 and 1. The static policy means that the motors are not activated at all and the arm does not move by itself. These policy combinations, although not exhaustive, represent a variety of different scenarios an agent can face in this setting. Haptic signals are used as the ground truth to determine which arms push the ball in each trial. Each scenario was executed for 100 trials, with the policies alternating between the arms each trial. The reports of each measure between the six SIPs of the arms and the SIP of the object were recorded and compared to the ground truth. As the accuracy score does not take into account false positives, and the previous set of experiments on the synthetic signals show that all the measures tend to report false positives in random signals, the F1 scores are used for the assessment. The F1 scores of the measures for each scenario are presented in Table 1.

**Table 1:** F1 scores of Acceleration Transfer (AT), Granger Causality (GC), and Transfer Entropy (TE) measures on seven mixed policy scenarios.

	**Scenario 1**	**Scenario 2**	**Scenario 3**	**Scenario 4**	**Scenario 5**	**Scenario 6**	**Scenario 7**
AT F1 Score	0.91	0.91	0.9	0.89	0.78	0.91	0.77
GC F1 Score	0.67	0.41	0.16	0.27	0.56	0.21	0.17
TE F1 Score	0.91	0.84	0.48	0.8	0.68	0.82	0.69

The F1 scores in Table 1 show that GC fails to correctly identify the direction of the causal relationships, especially with complex policies, reliably. This result is in line with expectations, as the setting pushes the limitations of the GC measure, explained at the beginning of the current section. TE and the AT measure successfully capture the directional causal relationships between the SIPs of the arms and the ball. AT keeping a stable performance across the first four scenarios where one arm is static, while the TE has decreased performance as the policy of the pushing arm gets more complex. Both measures have decreased performance in Scenarios 5 and 7, the only two scenarios where the arm that is not pushing the ball can have positive and negative acceleration and thus lead the measures to false positives as shown in the last signal in Figure 4.

In conclusion, correlational analysis of the SIPs can be used to characterize the self vs. the other and capture proximo-distal arm-joint dependencies and motor-sensory influences but is not sufficient to infer autonomy. TE, and the novel AT measure, but not GC, can reliably capture directional causalities between SIPs in this setting.

## 4. Discussion

We discuss, in this study, the minimal requirements for self/other distinction that lead to simpler methods in comparison with the approaches that require a multitude of parameters (such as deep recurrent neural networks Rabinowitz et al., 2018) or rely on the predictive coding hypothesis which requires a forward model to operate (Fairhurst et al., 2019). The typical approach to address the problem of self vs. other distinctions is based on the mismatch of the predicted perceptual state (given the precedent executed action) and the actual perceived state. A smaller mismatch of the (feedforward) prediction with the actually perceived state (feedback) would imply an increased degree of agency as stated by the *Comparator Model* (Wolpert and Flanagan, 2001; Farrer and Frith, 2002; Fairhurst et al., 2019).

We argue for the assumption that the ideal time for the distinction of self and other in early development to occur is prior to the acquisition of forward models, to be able to focus the learning and reduce the input dimensionality of the forward models to be learned. Following this assumption, we address the self and other distinction with the simplest method possible, a simple correlational analysis of visual movement features and in the absence of a forward model (Wolpert and Flanagan, 2001), or one could say that it is a forward model of purely visual features (as the introduced sensory to sensory predictions Maffei et al., 2017) and in a preliminary stage of learning to control. This preliminary learning phase is important as it can guide and reduce the dimensionality of the inputs to the more complex forward models (including motor signals) that subsequently need to be learned.

To clarify this simple mechanism, let us consider the following thought experiment. When playing a video game for the first time, we face a completely novel situation in which a forward model is not available. However, we can quickly discern which entities we are in control of by pressing buttons randomly, and we can direct attention to the immediate surroundings of those entities and reduce the state space dimensionality of the learning. In addition, imagine after having learned to control a character, we invert the SM mapping (pressing right makes the agent go left and vice-versa). The forward model that was acquired now makes wrong predictions (and needs to be relearned) but the feeling of self and other remained intact and has not been disrupted. Our approach, in this study, builds on Sánchez-Fibla et al. (2017b) and addresses exactly this preliminary stage.

We test this *via* a statistical and information theoretical analysis of the SM data stream (as performed in Hoffmann, 2014). The results show that the self/other distinction can be addressed solely by a correlational analysis of motor signals and their sensory effects (channeled through the motion, velocities and accelerations of the previously mentioned SIPs) prior to the construction of a forward model. For assessing autonomy, we need to go beyond correlation and perform a causality analysis to be able to extract directional dependencies. A rectified subtraction of feature accelerations, denoted in this study as AT, is shown to be sufficient to extract directional dependencies and as a cheaper alternative to more computationally expensive measures such as TE.

Autonomous motion alone is not the only information that infants use to assess agent/object autonomy as they check for autonomous control over the actions of entities' (Baillargeon et al., 2015). Considering the latter, there is a need to build forward models and the aforementioned *Comparator Model* applied to the assessment of control of other entities could be an explanation behind figuring out autonomy in its final phase. But before that, building a hierarchical structure of visual motion cues based on velocity correlations and AT may be a prerequisite step, as we have shown. From a neurophysiologycal perspective, there is evidence that there are neurons (in the brain MT area of the Macaque monkey) that are tuned to acceleration changes (Schlack et al., 2007).

## 5. Conclusions

From early development, self and other distinctions are fundamental to the focus the learning (reduce state space dimensionality) of forward model acquisition. From a computational perspective, discriminating between self and other features from visual feedback is often addressed through models (Brody et al., 2017; Sánchez-Fibla et al., 2017b; Thomas et al., 2017; Pertsch et al., 2018; Rybkin et al., 2018), which either require a multitude of parameters (deep learning approaches like Rabinowitz et al., 2018) or rely on the predictive coding hypothesis, requiring a forward model to be able to check the matching between current and predicted states (Fairhurst et al., 2019). In this study, we have approached this problem from a principled perspective, identifying minimum requirements to solve the problem of deciphering which features of the visual scene correspond to the self and which of them correspond to other entities in the scene, *via* a correlation analysis of velocity signals, that we have found to be sufficient. Thus, self/other distinctions could be identified with minimal and simpler methods, prior to the acquisition of the forward models and could guide and reduce the dimensionality of their inputs.

We do not work with images directly. Elements of the visual scene are interpreted *via* the SIPs, visual features (that we introduced in Sánchez-Fibla et al., 2017a, reminiscent of BM pivots that can be computed by computer vision methods like SIFT features). SIPs can be characterized as belonging to oneself thanks to the high motor to sensory correlations. Furthermore, from the full correlation matrix, proximo-distal joints can be characterized and also other entities with similar correlation patterns with their proximo-distal structures.

We go beyond the distinction of self and other by defining autonomy as the ability to move by oneself (animacy), and we discuss how we can detect it from visual SM interactions, a problem that has not been addressed yet, to our knowledge, from a computational perspective. For detecting autonomy, correlation comes with its limitations: it only captures linear relationships and does not allow us to infer directed/causal dependencies. To surpass this limitation, we looked at measures, such as GC, Copula-Based (Junker et al., 2019) and TE (Vicente et al., 2011), but we concluded that they were unable to capture directional dependencies on a trial-by-trial basis. Instead, they work better on distribution of sampled SM data. We grounded dependency assessment on a simpler principle of energy transfer between entities (energy in terms of acceleration). For this purpose, we developed a novel AT measure, that is not temporally dependent, and computed the estimated instantaneous transfer of acceleration (note, as we have discussed, that instantaneous effects pose problems for standard measures, such as GC) between two moving entities (in our case SIPs). The proposed AT measure works under the principle that an inanimate entity is always the sink in the directed SM graph of transfers and produces better results than the standard causality algorithms, but further research might compare other causal inference approaches (as discussed in section 3.3). AT would be a very natural way to interpret interactions between visual pivots as acceleration is proportional to force *via* Newton's 2nd law. In addition, neurophysiology findings back this hypothesis with proof of the existence of neurons with responses that are tuned to acceleration and deceleration (Schlack et al., 2007).

We have identified the minimal principles that we hypothesize are at play when making sense of embodied SM visual experiences, and we make a concrete proposal of what is the minimal level at which (causal) directional reasoning is needed to understand visual motion pivot interactions [as discussed in the challenges exposed in Pezzulo et al. (2011)]. Beyond the self/other distinction, which we have shown can be assessed by a correlational analysis of velocities (without the need for directional reasoning), we show that for the assessment of autonomy, directional inferences need to be utilized. We also hypothesize that these findings, grounded on developmental psychology, could also be transferred to developmental robotics (Cangelosi and Schlesinger, 2015). We also argue that transfer learning, that is the generalization of acquired knowledge from one task to another cannot be achieved without this fundamental step, by annotating the SM memory of the agent with “who did what and when.” As proof of concept, we refer to the results obtained in the article by Demirel and Sánchez-Fibla (2019), where a reinforcement learning agent speeds up its learning by having access to the features it controls from its perceptual state. In this sense, approaches based on cognitive architectures will require this information in SM memory. Up until now, this has been lacking in the proposed architectures (refer to the mentioned DAC framework Duff et al., 2011 and the SM graph structures introduced in Toussaint, 2006).

## Data Availability Statement

The datasets presented in this study can be found in online repositories. The names of the repository/repositories and accession number(s) can be found below: https://github.com/santmarti/PythonRobot2DSim.

## Author Contributions

All authors listed have made a substantial, direct and intellectual contribution to the work, and approved it for publication.

## Conflict of Interest

The authors declare that the research was conducted in the absence of any commercial or financial relationships that could be construed as a potential conflict of interest.

## Publisher's Note

All claims expressed in this article are solely those of the authors and do not necessarily represent those of their affiliated organizations, or those of the publisher, the editors and the reviewers. Any product that may be evaluated in this article, or claim that may be made by its manufacturer, is not guaranteed or endorsed by the publisher.

## Footnotes

^1^The simulation can be downloaded from https://github.com/santmarti/PythonRobot2DSim. A video TwoArmSetup.mp4 is also available under the folder videos in the same github repository. Notebooks for the generation of figures of the paper will also be made available.

^2^For GC we used the “statsmodels” Python module and for Transfer Entropy the Python interface to the Java Information Dynamics Toolkit.
